# Diet-dependent gene expression highlights the importance of Cytochrome P450 in detoxification of algal secondary metabolites in a marine isopod

**DOI:** 10.1038/s41598-018-34937-z

**Published:** 2018-11-14

**Authors:** Pierre De Wit, Keith Yamada, Marina Panova, Carl André, Kerstin Johannesson

**Affiliations:** 1 University of Gothenburg, Department of Marine Sciences, Tjärnö, Sweden; 2 University of Turku, Department of Biochemistry, Turku, Finland

## Abstract

Isopods of the genus *Idotea* have an unusual ability to feed on algae containing high amounts of chemical defense molecules, such as species of the genera *Fucus* and *Ulva*. In this study, we compared gene expression patterns of *Idotea balthica* individuals fed with *Fucus vesiculosus* to individuals fed with *Ulva lactuca*. We generated the first-ever transcriptome assembly for this species, and found 3,233 differentially expressed genes across feeding regimes. However, only a handful of biological functions were enriched with regard to differentially expressed genes, the most notable being “alkaloid metabolic process”. Within this category, we found eight differentially expressed cytochrome P450 (CYP) unigenes, all of which had a higher expression in the *U. lactuca* diet treatment. A phylogenetic analysis showed that the differentially expressed CYP genes are closely related to a CYP gene described from the hepatopancreas of the spiny lobster *Panulirus argus*, and we hypothesize that these transcripts are involved in metabolite detoxification. This is a first step in the understanding of this algae-grazer interaction, and will form a basis for future work to characterize cytochrome P450 functioning in marine crustaceans.

## Introduction

Isopod crustaceans of the globally distributed genus *Idotea* are common in coastal marine areas. These species are generalist grazers on macroalgae and sea grasses, and can be ecologically important in structuring algal communities^1^. For example, in some areas in the Baltic Sea (Northern Europe), densities of *Idotea balthica* can rise to astonishing numbers (>80 individuals/100 g wet weight of the brown alga *Fucus vesiculosus*), which may temporarily cause a complete loss of *F. vesiculosus* in the area^2^. In areas with strong grazing pressure, algal genotypes with defensive strategies against grazing are favored by natural selection^3^. One common defense strategy among macrophytes is synthesis of secondary metabolites, which are bioactive compounds not directly related to growth or reproduction. In algae, these are often involved in grazer deterrence by decreasing the palatability of the tissue^4^. As some algal secondary metabolites have been found to have pharmaceutical applications, much research has been undertaken to gain a better understanding of these compounds^5–9^. As a result, a whole range of bioactive compounds have now been discovered in seaweeds^10^.

However, the mechanism involved in grazer deterrence has been more difficult to conclusively elucidate to date. It has been suggested that the main grazer-deterring compounds in brown algae are polyphenolic compounds which have been named “phlorotannins”^11,12^. These compounds have the ability to bind to proteins and thus reduce the digestible nutrient content of the algae, while at the same time degrading the chemical environment within the gut of the grazers. Jormalainen *et al*.^13^ observed that phlorotannin extracts reduced the assimilation efficiency of *I. balthica*, but this did not deter the isopods from feeding. Other feeding trials with algae of varying phlorotannin concentrations have yielded mixed results, in some cases deterring grazing while in some cases actually stimulating feeding^14^. In green algae, such as the “sea lettuce” *Ulva lactuca*, dimethylsulfoniopropionate (DMSP) and acrylic acids^15^, as well as compounds producing reactive oxygen species (ROS)^16^ are induced under grazing, and have been suggested to have a role in grazer deterrence and toxicity. ROS ingestion could cause oxidative damage in the guts of grazers, as has been shown in insects feeding of plant tissue^17^. However, while chemical defense compounds found in *Ulva* species caused sea urchin deterrence, they had the opposite effect on *Idotea wosnesenskii*^15^. In common periwinkles (*Littorina* spp.), a recent study found that the deterrence effects of *Ulva lactuca* were species-specific, which the authors hypothesized could be due to differences in feeding strategies (generalist vs specialist)^18^.

As a consequence of the evolution of chemical defense compounds in the algae, it is expected that the grazers would evolve mechanisms for tolerance or detoxification of the algal compounds. A tolerance mechanism could act in different ways. For example, a highly efficient general detoxification mechanism for toxic compounds can be fine-tuned based on the diet. This would allow them to be generalists while still feeding on algal species with high quantities of secondary metabolites such as *F. vesiculosus* and *U. lactuca*. Common to all animals, the Cytochrome P450 (CYP) system is one of the major players in metabolism and detoxification of compounds produced by other organisms^19–21^. Cytochrome P450 is a superfamily of enzymes, consisting of several hundreds of different types which are classified into different families^22^. Some of these have an important role in general metabolism and in development, while others are more involved in detoxification^23^. For example, CYP enzymes are induced in caterpillars after being fed with nicotine-containing material^24^. In the marine environment, it has been shown that some variants of the CYP2 family, involved in detoxification of xenobiotic compounds, are present in the hepatopancreas of crustaceans^25,26^. While very few comparative studies have been conducted to date on crustacean CYP variants, it has been suggested that generalist algal grazers would benefit from the evolution of a high diversity of CYP genes^27,28^, as has been observed in the genome sequence of the purple urchin (*Strongylocentrotus purpuratus*)^29^. Each of these could then evolve an ability to metabolize a specific group of compounds, generating a finely-tuned diet-based detoxification machinery. In addition, it is possible that the isopods have acquired specialized gut microbiota, which assist them in digesting toxic compounds before they are able to diffuse from the gut into the tissues of the animal. In terrestrial isopods with diets containing hard-to-digest compounds such as cellulose, it is well-known that the gut microbiota plays an important role in digestion^30,31^. The functional roles of gut microbes in species of the genus *Idotea* have not yet been investigated, but there have been some studies of the overall microbiota indicating that isopods from different geographic areas and with different diets possess distinct microbiomes^31,32^. It is also possible that some of the functions usually performed by gut bacteria could have been transferred to the isopods themselves. For example, the lignocellulose metabolic machinery otherwise only present in bacteria and fungi, can be found also in the genomes of isopods of the wood-boring genus *Limnoria*^33^. In particular, the glycosyl hydrolase family 7 (GH7) has 3 representatives which are all expressed in the gut transcriptome of *Limnoria quadripunctata*^34^ suggesting horizontal gene transfer from symbiont to host^33^.

We here present a study investigating the detoxification mechanism of algal defense compounds in *I. balthica* using an RNA sequencing approach. We conducted an experiment where individuals of *I. balthica* were fed with different algal diets - one brown alga (*F. vesiculosus*) which is a common diet item for these isopods, and one green alga (*U. lactuca*) which is not as common in the isopods’ habitat. Representing two evolutionarily distant groups of algae, it is likely that there are considerably differences in metabolite structure among the two. Subsequently, we examined differences in gene expression among individuals from the different diet treatments supported by a reference transcriptome assembled from a combination of all tissue types from one adult male *I. balthica*. Our hypothesis was that the *I. balthica* individuals would induce expression of diet-specific detoxification enzymes, either through isopod-specific detoxification compounds, or as a response within the Cytochrome P450 pathway. If the latter was the case, we predicted a diversity of different Cytochrome P450 forms in the transcriptome sequence of *I. balthica*, of which many would be differentially expressed in different diet treatments, and that these would be similar in sequence to variants of Cytochrome P450 found in other marine algal grazers.

## Results

### Transcriptome Assembly and Annotation

The total number of reads amounted to 296,173,922, out of which 262,020,548 remained after quality-trimming. Rcorrector was able to correct 46,535,134 bases pre-assembly, and the *in silico* normalization yielded a final set of 10,099,252 read pairs which were used as input for the *de novo* assembly (Table 1).

**Table 1:** Assembly statistics.

Total Trinity unigenes	30,147
Total Trinity transcripts	40,122
Percent GC	39.7
N50	1,710
Shortest contig length	201
Median contig length	900
Average contig length	1,184.48
Longest contig length	21,522
Total assembled bases	47,523,534
Complete single-copy BUSCOs	762
Complete duplicated BUSCOs	378
Complete triplicated BUSCOs	185
Fragmented BUSCOs	320
Missing BUSCOs	1,030
Putative peptides	21,439
PANNZER annotation	10,655
BLASTx to nr (e < 10^−5^) annotation	19,498
Term-filtered	6,572
Microbe-filtered	5,875
Total annotated	12,924

The raw assembly was filtered using read evidence and presence of ORFs using TransRate, with a resulting output assembly of 40,122 transcripts in 30,147 unigenes, an N50 of 1,710 bp and a total length of 47,523,534 bp (Table 1). Mapping the reads of the reference individual back to the filtered assembly, 67% of the reads mapped (58% with high quality). TransDecoder could find 21,439 putative peptides encoded by the filtered assembly (Table 1). The BUSCO analysis searched for 2,675 Arthropod single-copy orthologs in the assembly, and found 1,645 (61.5%). Only 46.3% of these were single-copy and complete, and the remainders were fragments (19.5%), duplicates (23.0%) or triplicates (11.2%) (Table 1), resulting from either assembly fragmentation or separate assembly of the two alleles.

The PANNZER annotation, using an arthropod database, yielded annotations for 10,655 out of the 40,122 transcripts, while a separate BLASTx to NCBI’s non-redundant protein database (nr) yielded annotations for 19,498 transcripts (Table 1; Supplementary Table 1). Filtering out putative, hypothetical and predicted hits, this number was reduced to 6,572 BLASTx-annotated transcripts. Finally, BLAST hits from the most common micro-organismal genera were filtered out, leaving 5,875 transcripts annotated by BLAST. In total, 12,925 transcripts were annotated by BLAST and PANNZER.

The sequences of the *Limnorea quadripunctata* lignocellulose digestion genes originating from microbes (GH7 and GH9) had no BLAST matches in the *I. balthica* transcriptome.

### Feeding Trials

The sequencing depth ranged between 21,541,785 and 48,180,703 reads per individual (mean = 38,037,887 reads, st.dev. = 5,937,275 reads) (Supplementary Table 2). The proportion of reads that aligned to the reference transcriptome was between 61.1% and 88.6% (mean = 81.3%, st.dev. = 8.73%). Out of these, between 55.1% and 73.9% mapped to multiple locations in the assembly (at the transcript level) (mean = 65.1%, st.dev. 6.37%).

No reads were found to map to *I. chelipes* and *I. granulosa* COI sequences, whereas many (mean = 74,581, st.dev = 59,750) mapped to *I. balthica* COI, showing that all experimental individuals were indeed *I. balthica*.

The RSEM analysis of gene expression extracted expected count data, averaged by transcript/isoform for each unigene (Supplementary Table 3). The expected counts were rounded to the nearest integer and analysed in DESeq2, resulting in 3,233 genes being differentially expressed between feeding treatments (*Fucus* or *Ulva*) after multiple test adjustment (p < 0.05) (Supplementary Table 4). Out of these, 1,145 could be annotated (924 by PANNZER, 547 by BLASTx to nr) (Supplementary Table 5). Of these, 15 could potentially be microbial (or have isoforms within the “gene” which were microbial), as suggested by the BLASTx to nr taxon-filtering, although 2 out of the 15 did have a valid non-microbial PANNZER annotation (TRINITY_DN73620 (TEM beta-lactamase) and TRINITY_DN93555 (T-complex protein 1 subunit delta)) (Supplementary Table 5).

The multiple-test adjusted p-values from the differential expression analysis were used as input for GO enrichment Gene Score Resampling analysis. Despite the large number of differentially expressed genes (3,233), only 2 functional categories were significantly enriched for differential expression. Those were “nicotine metabolic process” (GO:0018933, adjusted p-value 2.01 * 10^−9^) and “alkaloid metabolic process” (GO:0009820, corrected p-value 4.02 * 10^−9^) (Supplementary Table 6). Nicotine metabolic process is nested within alkaloid metabolic process, and all genes in the former are also present in the latter. There are 16 genes responsible for the enrichment (Table 2), most of which are various forms of Cytochrome P450. All of the significantly differentially expressed genes in the list were down-regulated in the *F. vesiculosus* diet treatment. Quantitative PCR of three representative Cytochrome P450 genes confirmed this result (Supplementary Figure 1).

**Table 2:** Transcripts responsible for the functional enrichment of GO category “alkaloid metabolic process” (GO:0009820, corrected p-value 4.02*10^−9^).

Contig	Annotation			Mean Gene Expression	log two-fold change	Multiple-test corrected p-value
	nt	nr	PANNZER			
*TRINITY_DN87011*	*Cytochrome P450 2U1-like protein (Fragment)*	*Cytochrome P450 2L1*	*Cytochrome P450 2U1-like protein (Fragment)*	*3 012.6*	*−3.36*	*4.54E-07*
*TRINITY_DN93751*	*Cytochrome P450 CYP2*	*no annotation*	*no annotation*	*1 118.1*	*−3.13*	*5.29E-05*
*TRINITY_DN78647*	*thromboxane A synthase*	*thromboxane A synthase*	*Cytochrome P450 3 A*	*183.0*	*−2.25*	*3.95E-04*
*TRINITY_DN91636*	*Cytochrome P450 family 2 subfamily B*	*Cytochrome P450 2L1*	*Cytochrome P450 family 2 subfamily B*	*1 789.3*	*−1.32*	*1.06E-03*
*TRINITY_DN91758*	*nicotinamide phosphoribosyltransferase*	*no annotation*	*no annotation*	*458.2*	*−0.55*	*1.39E-03*
*TRINITY_DN92128*	*Cytochrome P450 2J2*	*no annotation*	*no annotation*	*356.0*	*−1.92*	*1.71E-02*
*TRINITY_DN91434*	*Cytochrome P450 2C42/ADP ribosylation factor*	*no annotation*	*no annotation*	*527.8*	*−1.23*	*2.04E-02*
*TRINITY_DN89473*	*Cytochrome P450 2K4*	*no annotation*	*no annotation*	*603.9*	*−1.54*	*4.42E-02*
TRINITY_DN91160	no annotation	no annotation	no annotation	1 105.6	−0.78	5.81E-02
TRINITY_DN93784	senecionine N-oxygenase/flavin-containing monooxygenase	no annotation	Flavin-containing monooxygenase FMO GS-OX-like 8	499.3	−0.70	6.01E-02
TRINITY_DN93543	no annotation	cytochrome P450 monooxygenase	Cytochrome P450, family 2, subfamily V, polypeptide 2	357.8	−1.02	6.43E-02
TRINITY_DN92806	Cytochrome P450 2C3/2K4	unnamed protein product, partial	no annotation	623.9	−1.18	6.56E-02
TRINITY_DN93351	tumor necrosis factor alpha/retinol dehydrogenase/cytochrome P450	no annotation	Cytochrome P450 CYP2N	939.5	−0.97	6.67E-02
TRINITY_DN92833	Unknown function, Nematode genome assembly	cytochrome P450 CYP6B53	Cytochrome P450 3 A (Fragment)	15.5	−1.78	1.21E-01
TRINITY_DN92074	Cytochrome P450 9e2	cytochrome P450	Cytochrome P450 9e2	5.6	−0.70	4.74E-01
TRINITY_DN92058	dopa carboxylase	Aromatic-L-amino-acid decarboxylase	Aromatic-L-amino-acid decarboxylase	16.6	0.57	4.93E-01

In italics are significantly differentially expressed (corrected p < 0.05) transcripts.

**Figure 1:**
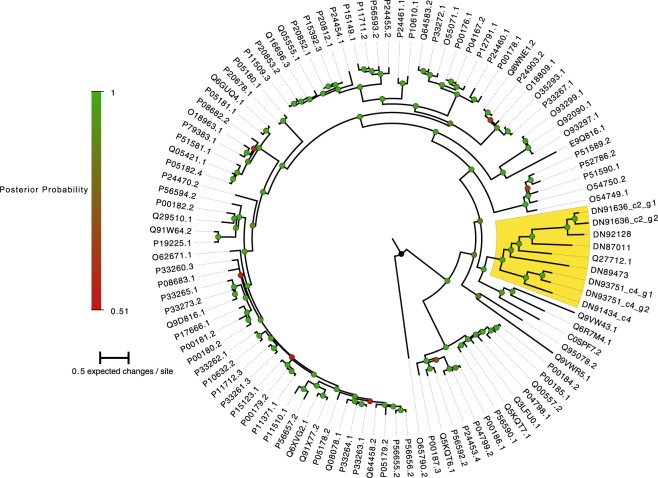
Phylogenetic tree of eight *I. balthica* CYP450 transcripts (DN######), together with 100 CYP450 sequences downloaded from GenBank (See Supplementary Table 1 for a list of Accession Numbers). Node support values (Bayesian posterior probabilities) are denoted by the coloured circles. The clade grouping all *I. balthica* transcripts together with a *Panulirus argus* transcript (Q27712.1) is highlighted in yellow.

The differentially expressed Cytochrome P450 isoforms were merged by sequence identity into 8 different sequences, and a BLASTp search using the ORF protein sequences showed that all belong to the CYP2 family. The results of the Bayesian phylogeny inference showed that all 8 sequences form a monophyletic clade together with Cytochrome P450 2L1, described from the hepatopancreas of the spiny lobster *Panulirus argus*, with strong statistical support (Q27712.1; Fig. 1). In addition, one of the contigs with Cytochrome P450 domains also contained components with ADP-ribosylation domains (TRINITY_DN91434_c7). There was only one differentially expressed unigene in the GO category (TRINITY_DN91758) which was not annotated as a Cytochrome P450, but rather as a nicotinamide phosphoribosyltransferase by BLAST to the nt database (Table 2).

## Discussion

The Cytochrome P450 family 2 is specifically involved in detoxification of xenobiotic compounds by mono-oxygenation or hydroxylation, thereby inactivating potential bioactive compounds which have been ingested^35^. Usually, CYP detoxification involves additional binding proteins such as NADP+, which can act as electron receivers^36^. The CYP2 transcripts found in this study are most closely related to those described from the hepatopancreas of spiny lobsters (CYP2L^25^). However, to the best of our knowledge, no work has been done on the actual mode of detoxification within crustaceans, and it is not known which specific compounds these CYP2 proteins might be metabolizing. Further work is thus essential here to investigate these genes and their functions.

Nicotinamide phosphoribosyltransferase (NAMPT) was the only non-CYP2 unigene within the functionally enriched categories in our experiment. NAMPT plays a critical role in production of NAD+^37^, which has an important role in metabolism via the electron transport chain. It has recently been shown that a knockout of NAMPT has a cascading effect on the cytochrome P450 pathway and inducing mortality in mice^38^, suggesting that NAMPT is needed for the CYP2 proteins to function.

Interestingly, all of the CYP2 transcripts were more highly expressed in *I. balthica* fed with *U. lactuca* than in isopods fed with *F. vesiculosus*. Several recent studies have shown that *Ulva* produces a range of secondary metabolites which deter both gastropods^18^ and sea urchins^39^. *F. vesiculosus* also possesses a range of secondary metabolites, but as *F. vesiculosus* is the natural diet of *I. balthica*, we can speculate that they might be able to detoxify *F. vesiculosus* compounds through a separate, taxon-specific, pathway. As support of this hypothesis, our results indicate that there are many unannotated transcripts which are upregulated in the *F. vesiculosus* diet treatment. Some of these could be involved in detoxification. An alternative explanation could perhaps also be found in that *F. vesiculosus* possesses a structural defense in its much more robust and leathery thallus, which might relax the need for production of secondary metabolites aimed at grazer deterrence, when compared to *U. lactuca* which has a thin and soft thallus.

In isopods of the genus *Limnoria*, lignocellulose metabolic genes (primarily GH7) have been horizontally transferred from bacteria to the isopod, allowing them to digest plant (wood) material without symbiotic gut microbes^33^. In the *I. balthica* transcriptome assembled in this study, we found no signs of GH7 homologs. Perhaps this is not surprising, as genes involved specifically in lignocellulose metabolism might be irrelevant to grazers of lignocellulose-lacking algae, such as *I. balthica*.

For future work, proteomic and metabolomic tools could help us understand how metabolite concentrations change with varying transcript levels. The link between transcript expression and physiological performance is not always straightforward due to e.g. non-linear relationships between transcript and protein expression and/or between protein expression and activity^40^, so gene expression studies such as this one can only elucidate one piece of a more complex puzzle.

In conclusion, our data shows that expression of the Cytochrome P450 family 2 is influenced by the species of macroalgae that *I. balthica* is feeding on. As this is a gene family involved in detoxification, this suggests that these genes may be important in the isopod detoxification of secondary metabolites produced by the algae, and that there is a diet-specific regulation at the individual transcript level. It would be of great interest to further investigate CYP gene family evolution in *I. balthica*. However, in order to do this, a reliable genomic reference sequence will be required. Until then, we can conclude that the picture is complex and more work will certainly be needed before the mechanisms of metabolite detoxification in marine grazers can be fully understood.

## Materials and Methods

**Figure 2:**
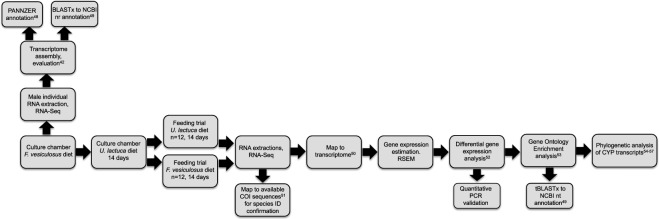
Flowchart of the RNA-Seq analysis undertaken in this study.

The analysis followed a standard RNA-seq pipeline, similar to the one published by De Wit *et al*.^41^ (Fig. 2). Below are detailed Methods for each of the steps listed in the flowchart.

### Transcriptome Assembly and Annotation

The male *I. balthica* individual from which the transcriptome was assembled was sampled in September, 2014 at Kristineberg, Sweden (58°14.869′N;11°26.883′E). Total RNA was extracted using a TRIzol (Thermo Fisher Scientific) protocol and cleaned with a Zymo RNA Clean and Concentrator Kit (Zymo Research). RNA concentration was measured using a QuBit RNA assay (Thermo Fisher Scientific) and RNA integrity was assessed using a MOPS denaturing agarose gel. cDNA libraries were prepared at the National Genomics Infrastructure in Stockholm, Sweden using a TruSeq RNA Library Preparation Kit v2 (Illumina). This protocol includes poly-A selection of mRNA, cDNA synthesis, Illumina adapter ligation and a 12-cycle PCR reaction. A single lane of an Illumina HiSeq2500 sequencer (Illumina) was then used to paired-end sequence 125 base pair (bp) long reads.

The ‘best practice’ transcriptome assembly pipeline by De Wit *et al*.^42^ was followed to generate a total male transcriptome. All bioinformatic analyses were run on the CSC - IT Center for Science’s (Espoo, Finland; www.csc.fi) Taito super-cluster. Briefly, raw reads were assessed for quality using FASTQC (v0.11.2; www.bioinformatics.babraham.ac.uk/projects/fastqc), after which TRIMMOMATIC (v0.33)^43^ was used to trim adapters and trimming read ends for low-quality bases. Also, a sliding window of four bases was used for trimming reads of average base quality <15. Finally, sequences that were less than 36 bases long were dropped.

Pre-assembly error correcting was done using RCORRECTOR (v2015-11-06^44^) with a k-mer size of 20 to reduce the number of substitution errors incorporated into the assembly. TRINITY (v2.1.0) was used for *in silico* normalization^45,46^ with k-mer size 25 and a coverage limit of 50. The reads were then *de novo* assembled using TRINITY with a k-mer size of 25.

The assembly was quality evaluated and filtered using TRANSRATE (v1.0.1^47^). The normalized reads were used for read-mapping. Only contigs with read evidence and containing ORFs were kept. The filtered assembly was assessed for the presence of putative peptides by TransDecoder (available at http://transdecoder.github.io/). The raw data used was submitted to the NCBI Sequence Read Archive (SRA) and the final version of the assembly was submitted to the Transcriptome Shotgun Assemblies (TSA) database (BioSample SAMN08960750).

A BUSCO analysis was run to evaluate the completeness of the transcriptome assembly with respect to the number of known core single-copy orthologous genes identified^48^. In this study, BUSCO was run in transcriptome mode using the arthropod lineage dataset as a reference. In cases where orthologues were found to be duplicated in the assembly, the exact number of times they were found was determined using a custom script.

Annotation was conducted in two separate ways. Firstly, functional annotation was conducted using PANNZER^49^. PANNZER searches the UniProtKB/Swiss (http://www.uniprot.org) database and uses a Z-score for a more reliable automated annotation^49^. The query taxon id 82763 (*Idotea balthica*) was specified to the software; the other settings were left as default. In addition, annotations were found using BLASTX (v2.2.31+^50^) against the NCBI non-redundant protein sequence database (nr; v2015-12-01) using an e-value cutoff of 10^−5^. Results were filtered to separate out uninformative terms (i.e. hypothetical, putative, predicted and unknown) using a custom script. The term-filtered output was then grouped by taxon using a custom script, after which bacterial and protozoan genera were filtered out. All scripts used can be found on GitHub (https://github.com/The-Bioinformatics-Group/Idotea_balthica_ transcriptome_project/tree/master/Annotation/blastx2nr/scripts).

Finally, in order to investigate the potential of horizontal gene transfer of lignocellulose digestion genes from microbes, as previously found in *Limnoria quadripunctata*, the glycosyl hydrolase (GH) family 7 and 9 members described in *L. quadripunctata* (GenBank Acc.Nos FJ940756– FJ940761) were BLASTed with a standard nucleotide BLAST against the *I. balthica* transcriptome assembly, using default settings.

### Feeding Trial Experiment

To find out which genes were differentially expressed under different feeding regimes, and if specific “detoxification genes” are activated in isopods when digesting algal metabolites, we performed a feeding experiment. Twenty-four individuals of *I. balthica* were collected at the same time and in the same location as the transcriptome individual (September 2014; 58°14.869′N;11°26.883′E). These isopods were kept in tanks with through-flowing seawater until the start of the experiment in the summer of 2015. Isopods were fed daily with their natural diet, the brown alga *Fucus vesiculosus* prior to onset of the experiment. At the start of the experiment, all individuals had their diet switched to *Ulva lactuca*, the “sea lettuce”, a green alga preferred by many marine invertebrates. After two weeks feeding on *U. lactuca*, 12 individuals were switched back to a *F. vesiculosus* diet. After another two weeks, all 24 animals were killed by decapitation and placed in RNAlater. (One individual in the *F. vesiculosus* diet treatment died prior to the end of the experiment and was excluded from further analyses). RNA was extracted and assessed as described above and cDNA libraries were prepared using the TruSeq RNA library prep kit v2 (Illumina). cDNA library fragment length was assessed using an Agilent TapeStation with a D1000 tape (Agilent), after which the fragments were multiplexed equimolarly (2 pools per treatment and 6 individuals per pool, except one pool which had 5 individuals) and were sent for sequencing at the National Genomics Infrastructure’s “SNP & SEQ platform” in Uppsala, using an Illumina HiSeq2500 machine (50 bp reads, single-end).

Sequence data were filtered for Illumina adapter sequences and low-quality ends (Q < 20) using fastx toolkit (available at http://hannonlab.cshl.edu/fastx_toolkit/). Filtered reads were then mapped to the transcriptome assembly using bowtie2 (v. 2.2.7)^51^, allowing for 5 mismatches, both with duplicate reads (two or more short reads with identical nucleotide sequences) kept and with duplicate reads removed, in order to assess duplication bias. As number of duplicates was highly correlated with expression values, and thus likely of biological relevance, duplicates were kept in downstream analyses.

Reads were also mapped (allowing for 1 mismatch) to a list of cytochrome oxidase subunit I (COI) sequences from all three *Idotea* species present in Swedish waters^52^, in order to ensure that all individuals were *I. balthica* (morphological species identification, in particular of juvenile individuals, can be challenging).

Gene expression values were obtained using the RSEM pipeline (https://github.com/bli25ucb/RSEM_tutorial). Briefly, the “–transcript-to-gene-map” option was first used to create a transcript to gene map of the transcriptome assembly, then raw expression values (expected counts) were extracted at the gene level. The expected counts were used as input for a differential expression analysis using DESeq2^53^, rather than FPKM values, as recommended by the manual. Finally, a GO enrichment Gene Score Resampling (GSR) analysis was performed on the multiple-test corrected p-values from the differential gene expression analysis in ErmineJ^54^. Genes responsible for GO categories being enriched for differential expression were, in addition to the annotation available for the entire transcriptome, also annotated by tBLASTx to the NCBI nt database.

In order to investigate the evolutionary relationships of differentially expressed Cytochrome P450 unigenes, 100 CYP protein sequences obtained from GenBank (Supplementaly. Table 7) were aligned to the translated *I. balthica* sequences, using Blosum62 cost matrix, and gap open/extension penalties 12/3 in Geneious 4.8^55^. The resulting protein alignment was examined for the best-fitting model of protein evolution using ProtTest 3^56^, after which the best model (JTT + I + G + F) was implemented in an MCMC phylogeny inference analysis using MrBayes v 3.2^57^. Two separate runs of 4 chains each were run for 11.5 M generations, sampling every 1000 generations and, after examining the output for convergence using RWTY in R^58^, 5 M generations were discarded as burn-in. Remaining generations were summarized into a majority-rule consensus tree and plotted using FigTree v1.4 (http://tree.bio.ed.ac.uk/software/figtree/). All raw data from the feeding trial experiment was submitted to SRA (BioProject PRJNA451082).

Finally, to validate our gene expression results, we performed quantitative PCR on three of the cytochrome P450 genes, using a subset of six individuals, three from the *F. vesiculosus* diet (T04, T05 and T06) and three from the *U. lactuca* diet treatments (C04, C06 and C12). Primers were designed based on consensus sequences from all isoforms of the CYP transcripts DN78647, DN87011 and DN89473, as well as 18S rRNA (DN93465), which was used as endogenous control (Supplementary Table 8). 0.5 µg RNA was converted to cDNA using an iScript select cDNA synthesis kit (Bio-Rad), after which a comparative C_T_ experiment using Fast SYBR Green (Thermo Fisher) was performed with an Applied Biosystems StepOnePlus system, following the default “fast” thermal program. The cDNA was diluted 1:10, and each reaction well contained 4 µl diluted cDNA, 1 µl primer F (10 µM), 1 µl primer R (10 µM), 4 µl nuclease-free water, and 10 µl SYBR Green master mix (x2), for a total reaction volume of 20 µl. Each reaction was replicated three times in a 96-well plate. In addition, each primer combination was also run in three negative control reactions. The sample T06 was arbitrarily set as the reference sample for the relative quantification.

## Electronic supplementary material

Supplementary Figure 1

Supplementary Table 1.

Supplementary Table 2.

Supplementary Table 3.

Supplementary Table 4.

Supplementary Table 5.

Supplementary Table 6.

Supplementary Table 7.

Supplementary Table 8.
