# Supplementary material for: Diet-dependent gene expression highlights the importance of Cytochrome P450 in detoxification of algal secondary metabolites in a marine isopod
Source: Sci Rep. 2018 Nov 14;8:16824. doi: 10.1038/s41598-018-34937-z (PMC6235865; doi:10.1038/s41598-018-34937-z)
Supplement: Supplementary file 1 — Supplementary Figure 1 [file 41598_2018_34937_MOESM1_ESM.pdf]

SUPPLEMENTARY INFORMATION FOR:

Diet-dependent gene expression highlights the importance of Cytochrome P450 in detoxification of algal secondary metabolites in a marine isopod

Pierre De Wit[1*], Keith Yamada[2], Marina Panova[1], Carl André[1] & Kerstin Johannesson[1].

[1] University of Gothenburg, Department of Marine Sciences, Tjärnö, Sweden.

[2] University of Turku, Department of Biochemistry, Turku, Finland.

[*] Correspondence to: pierre.de_wit@marine.gu.se

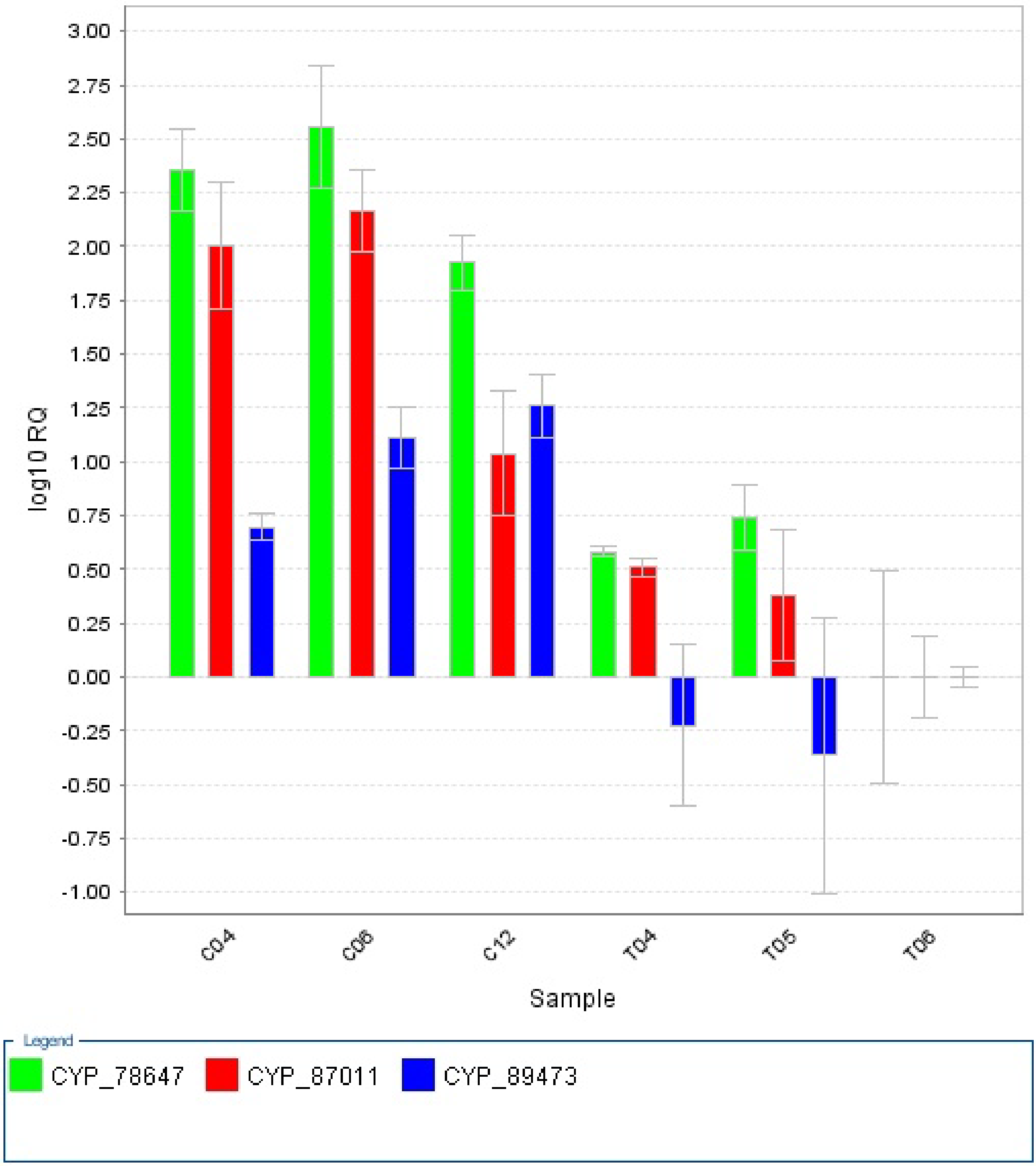

Supplementary Figure 1. Relative gene expression of three CYP genes. Sample T06 was arbitrarily set as reference for the relative quantification. Samples C04, C06 and C12 were fed *U. lactuca*, samples T04, T05 and T06 were fed *F. vesiculosus*. Error bars are SEM (n=3 for each sample). 18S rRNA was used as endogenous control.
